# Thoughtful Accompaniment in Life's Final Stages: Philosophical Practice as a Complement to Ethics Consultation

**DOI:** 10.1111/bioe.70031

**Published:** 2025-09-12

**Authors:** Patrick Schuchter, Sandra Radinger, Stefanie Veronika Rieger, Klaus Wegleitner

**Affiliations:** ^1^ Center for Interdisciplinary Research on Aging and Care (CIRAC) University of Graz Graz Austria; ^2^ Center for Applied Nursing Research University of Applied Sciences Campus Vienna Vienna Austria; ^3^ Institut für Pastoraltheologie und Pastoralpsychologie University of Graz Graz Austria

**Keywords:** assisted dying, ethics consultation, health sociology, medical ethics, philosophical consultation, philosophical practice

## Abstract

This paper argues that philosophical practice can complement existing medical ethics structures by offering a publicly accessible space for discourse and negotiation of basic concepts that are relevant to ethical decision making. The potential of collaboration becomes particularly evident by the example of assisted dying: it raises a wide range of philosophical questions which, however, tend to remain unarticulated where there is urgency for action and focus on ethical dilemma. In this paper, we first provide an analysis of the neglect of profound questions in ethics consultation and why these need to be considered and negotiated within a broader socio‐philosophical framework and place. These reflections are grounded in conceptual approaches informed by Immanuel Kant, Charles Taylor, and the tradition of Hellenistic philosophy as interpreted by Pierre Hadot. These frameworks are not presented as final answers, but as productive starting points and historically significant philosophical thought figures for engaging with the complex philosophical dimensions of assisted suicide. Then, we draw upon insights from an ongoing research project on philosophical practice in palliative care and hospice work. Interim results indicate that people experience a need for a kind of dialogue that gives such philosophical considerations a space and a person who is capable of facilitating it. Synthesizing theoretical‐philosophical and empirical insights, the paper provides an outlook for fruitful collaboration amongst medical ethics structures and philosophical practice.

## 1 Introduction

Addressing the question of the role of medical ethics expertise and ethics support in practices of assisted dying, in this paper, we focus on “ethics consultation” to illustrate the need for complementation by philosophical practice. Albeit this focus is a simplification of medical ethics structures, it suits the purposes of this paper as ethics consultation has become an established institution through which medical ethics expertise and support have a direct impact on practice.^1^ As more and more countries are decriminalizing assisted suicide, debates about ethically acceptable forms of assisted dying have (re‐)intensified. This challenges fields such as palliative care to reconsider so far supported positions [3]. In this paper, we point out that these debates lack explicit socio‐philosophical framing and a respectively accredited “place,” which are both needed to acknowledge and negotiate the societal impact on (decisions around) how we live and die. Contemporary debates and ethics consultation center mainly on legal regulations and moral questions regarding the specifics of implementation or non‐implementation in practice and within organizations. Simultaneously and implicitly, however, they also determine answers to fundamental philosophical questions, for instance, around conceptions of humanity, good life, freedom, and the boundaries of our actions between doing and letting be. We argue that philosophical practice can be a complement to ethics consultation in this regard. In this paper, we first provide an analysis of the neglect of profound questions in ethics consultation and why these need to be considered and negotiated within a broader socio‐philosophical framework and place. Then, we draw upon insights from an ongoing research project on philosophical practice in palliative care. These illustrate how philosophical practice provides the required socio‐philosophical framing and social space that can support people with developing positions toward fundamental issues around assisted dying. The paper closes with practical implications and conclusions regarding the need and potential of collaborative efforts of ethics consultation and philosophical practice.

## 2 The Philosophical Dimensions of Assisted Dying: Reclaiming a Missing Perspective

### 2.1 The Fate of Human Reason According to Immanuel Kant

In this paper, we draw upon Immanuel Kant's description of philosophical questions to better understand the demands that arise for ethics consultation around practices of assisted suicide in contemporary times. Kant's description of the “fate of human reason”—and, as discussed below, Taylor's analysis of the malaise of modernity along with the Hellenistic critique of representations—is not to be read as a fixed position or as a direct guide for individual decision‐making. Rather, these are robust and historically significant philosophical thought figures that serve as lenses through which we seek to approach assisted dying and the fundamental questions it raises.

Kant writes:

Human reason has the peculiar fate […] of being troubled by questions which it cannot reject […] but also cannot answer […] [4]

This thought describes the human situation as one fundamentally characterized by having to answer certain questions that cannot be resolved once and for all by human reasoning, yet are of such significance that they cannot be ignored either—on the contrary, our lives tend to revolve around them. Questions of that kind are, for example, if human will is truly free, whether there is an immortal soul, what constitutes a fulfilled life, and so forth. According to this perspective (as articulated by Kant), which we adopt as a point of departure for this contribution, such questions resist final or universal resolution; they cannot be conclusively answered at the level of general humanity. Yet, drawing on Plato, one might suggest that for individuals, responses may gradually take shape—ripening through lived experience and sustained intellectual exchange—until, perhaps, a moment of intuitive clarity emerges [5]. Even then, however, attained positions can never claim the status of absolute and universal validity.

We draw upon Kant's description of how human life is characterized by philosophical questions, because many questions surrounding (assisted) dying fall into that category. Evidence is provided, for instance, by Héctor Wittwer's comprehensive study, *Suicide as a Philosophical Problem*. Like the Socratic dialogues in Plato and pure reason in Kant, it ends in aporia: “A strict moral prohibition of suicide cannot be convincingly justified.”—“The permissibility of suicide cannot, however, be justified in terms of a strict principle either” [6]. So, (assisted) suicide demonstrates to be an existential and socially urgent question that can neither be dismissed nor answered in an absolute sense. At the social level, an important question arises: if ethics consultation is a social institution that supports people and organizations in resolving unanswerable philosophical questions around assisted dying, how do answers to and a handling of philosophical questions evolve at the societal level?

Kant describes three ways of how humans react to the impasse of reason surrounding philosophical questions, two of which he considers inadequate: dogmatism and a form of skeptical neutralization. The way forward, according to Kant, lies in striving for a “critical position”. In the following, we draw upon Kant's categories to analyze contemporary debates around practices of assisted dying and highlight a so far neglected demand for and potential of ethics consultation.

### 2.2 “Traditional” Dogmatism Versus “Modern” Skeptical Neutralization

In the debate on assisted dying, the “traditional” simplification of dogmatism seems to appear in two forms. There are persons and institutions who appear with possession of specific knowledge based on which they advocate that assisted suicide must “naturally” be either prohibited or allowed. Additionally, dogmatism manifests as an external attribution. The more discussions of assisted dying enter public media, the more opposing positions become ideologically labeled through simplistic attributions. This way, particular views are turned into a supposedly universal certainty, which does not do justice to the depth of the philosophical question of assisted dying nor to the nuanced perspectives and ways of life in a pluralistic society.

The “modern” simplification of skeptical neutralization proceeds in the opposite manner. It denies the possibility of a generalizable answer and “indifferently” leaves issues to individuals: if questions of what is good (life and death) cannot be resolved, each person must be allowed to decide for themselves as long as they do not harm others. This logic is effectuated by “the liberalism of neutrality” [7]. This idea manifests in legal frameworks of practices of assisted dying, whereby it is not surprising that recent constitutional rulings state that a general prohibition of assisted suicide contradicts individual freedom (e.g., in Germany or Austria). This way, assisted suicide and the philosophical questions it raises are not a subject of social, political, and legal discourses but of particular choices of persons or organizations.

The critical question for medical ethics and ethics support now is, whether there still is need for a social space and practice that supports the developing of answers to philosophical questions at the societal ethical level?

### 2.3 Philosophical Questions in the “Malaise of Modernity”

Acknowledging that the modern strategy of skeptical neutralization gives credit to the fundamental liberal constitution of societies, a critical perspective and acknowledgment of the “malaise of modernity” it brings about appears necessary too. Taylor's conceptualization of this discomfort [7] points out that developments and characteristics of modern societies are initially acknowledged as achievements and respectively defended. At a second glance, however, these characteristics tend to also reveal darker sides that pose real dangers to society if over‐emphasized. It is thus necessary to ask whether specific decisions and developments in society exacerbate or balance the malaise of modernity and the dangers it implies.

Regarding decisions and developments surrounding the philosophical question of assisted suicide, dangers can be identified around the tipping points of individualism and instrumental reason. Taylor first describes these as modern achievements: “individualism” is the emancipation of the individual subject from collective social structures and its elevation as the smallest unit of the social and legal order, whereby the individual is protected from coercion and paternalism of all kinds; “instrumental reason” is the essentially economic‐technological rationality as realized in scientific‐technical progress and capitalist growth economies. However, Taylor also names the points where these modern achievements shift from being institutions of problem‐solving to institutions of problem‐creation:

Individualism shifts from an achievement to a threat if the human image is reduced, if substantial relationships in public and communal life are lost, and if the concept of autonomy to self‐fulfillment assumes the trivial sense of an uncritical and immune‐to‐criticism demand for the realization of one's desires and ideas.

Instrumental reason shifts if the logic of technical control spills over into social, psychological, existential, and spiritual processes, and when an economic dynamic unfolds that increases growth without limits that is no longer beneficial for people and the planet but becomes an end in itself.

These tipping points are significant to the social organization of contemporary ethics consultation that operates primarily at the individual and organizational level. Individualism and instrumental reason are the guiding ideas of the practices and feelings of subjects and the modern social structures in which they are embedded. Moreover, in light of the discomfort with modernity, ethical considerations around assisted dying demand for shifts between questions of permission to questions of prudence: Are we, as a society, prudent enough to live individualism and instrumental reason in practices of assisted dying without falling into dogmatism and skeptical neutralization? Such questions have too little space within the currently dominant paradigms of medical ethics and ethics consultation.

What is needed in handling the philosophical question of assisted suicide at both the individual and societal levels seems to be forms and spaces of reflection that involve and enable processes of critical consideration of the main ideas of modern society. The following section suggests how such a critical consideration may look by example of a philosophical critique of conceptions that are relevant to the ethics of assisted dying.

## 3 A Philosophical Critique of Basic Conceptions Surrounding Assisted Dying

To acknowledge and live with the philosophical question of (assisted) suicide, we suggest with Kant a “critical way,” namely in the form of a philosophical critique of basic conceptions, as a third path between dogmatism and skeptical neutralization. In the antiquities, this approach was conceived of both as an individual art of living and as a political art of community, by Socrates, Epicurus, and the Stoics, for example [8, 9]. Situated within the very heart of everyday social life, rather than schools and universities, this take on the philosophical critique of basic conceptions appears most relevant to ethical considerations of good life and death. It builds on the following rationale:

1. “It is not things themselves that disturb people, but their conceptions of things” [10]. These conceptions are deeply “internalized” in us—in our feelings, spontaneous opinions, our “gaze,” and our daily social practice. The main philosophical problem is thus not constituted by the “thing” in question but potentially false or heavily one‐sided conceptions related to it.
2. These conceptions are socially and culturally constructed. What is considered “normal,” “right,” “healthy,” and so forth is defined within the context of socially permissible ways of living and thinking, within the framework of real and mental infrastructures.
3. This is why a critical reflection on both dominant and suppressed conceptions regarding relevant topics is needed.

Addressing the central question of whether current cultural and societal developments around assisted dying exacerbate the malaise with modernity or critically balance it, we now follow the path of a philosophical critique of basic conceptions. Note that the value we see in it does not rely on the assumption that the following matters are not considered in philosophical and ethical publications. The point is that communal philosophizing is not organized like ethical consultation in hospitals and nursing homes, structurally established with corresponding professional roles. We explicate a philosophical critique of basic conceptions surrounding assisted dying step by step because we see that society misses an institutionalized space and practice where not only a small group of experts but also ordinary people in their everyday lives can form their stance through critical discussion. In the case of assisted dying, these discussions may focus on philosophical critique of the following basic conceptions.

### 3.1 Critique of Conceptions of Humanity

Regarding the downsides of modern individualism, the notion of humanity seems most significant as it is implicit in all decisions surrounding assisted practices of human dying. At the most basic level, there are the questions of what is humanity and which conception of humanity is being quietly decided upon. Going into more detail, a key argument (in current jurisprudence) for the legalization of assisted suicide is the strengthening of individual autonomy. Does this already exaggerate the conception of the human “rational decision‐maker” that is already socially dominant? Or is it actually another necessary and logical step in the emancipation of the individual from paternalism? What appears relevant is that the strong, unclouded emphasis on the autonomous rational self tends to leave the bodily and emotional aspects of the self and the relational nature of autonomy out of sight. How can we uphold what may be seen as one of the greatest achievements of modern societies—the protection of individual autonomy—while avoiding an overemphasis which, as care ethicists [11] emphasize, cuts us off from being connected to our own embodiment and vulnerability, as well as from our connectedness to others?

### 3.2 Critique of Conceptions of the Good Life

The focus on the rationality of the healthy person in debates of practices of assisted dying might imply a tendency to further obscure the aspects and qualities of life phases where autonomy does not triumph [12]. If these and situations of vulnerability and dependence remain outside the image of the rational, autonomous self‐image, they are barely conceivable as anything other than an affront, humiliation, and shame. As a result, conceptions of good life that include dependency and neediness are devalued and those affected are excluded from “good life”. At the level of the mental infrastructure, the richness of human experience is reduced to what is complacent about the rationale, autonomous, healthy self. But who is to say that there are no important and valuable human experiences that lie in the vulnerable phases of life?

### 3.3 Critique of Conceptions of Freedom

Further connected to the malaise with individualism is the question of whether we risk trivializing the concept of freedom and endanger social cohesion by overemphasizing individual freedom. A tipping point of individualism in liberalism is that people lose a broader social view because freedom is focused on the individual and implies a narrow focus on the self [7, pp. 2–4]. This way, fundamental social and human questions are being excluded from public discourse and life forms immunized by a glorification of individualized lifestyles. If we open the concept of freedom to a relational one, however, the possibility of a “critique of life forms” [13] appears necessary and possible, allowing for reflection on the good life within a broader horizon of serious questions. In this regard, the critical question seems to be whether the concept of freedom that underlies current practices of assisted suicide strengthens societal cohesion and relationships, or amplifies a focus on the self? Here, it is worth considering whether we should prioritize a substantive concept of freedom over a formal one. As long as care, living with dementia, and accepting help remain “unimaginable” to the consciousness of the healthy, it is a legitimate question whether a decision for assisted dying can truly be considered an act of freedom—or whether it is simply an act of escape and avoidance of unpleasant questions and experiences life has to offer. We see with this question that the concepts of humanity, good life, and freedom are closely intertwined when it comes to ethical considerations of assisted dying.

### 3.4 Critique of Conceptions of Doing and Allowing

The final conceptions we see relevant to ethical considerations of assisted suicide and want to mention here are those of doing and allowing. They are relevant because, in medical ethics, instrumental reason is dominant. In this regard, we may ask: do legalization and acceptance of assisted suicide reinforce the mode of existence and worldview of the modern human in the form of technical control and domination over nature, or are they a necessary part of progress? Have we adequately practiced and integrated attitudes of allowing, letting‐be, and self‐limitation as individuals and as a society into our systems? If we take into account palliative care and hospice work, these have developed their own philosophy and culture as a response and counter‐model to the one‐sided dominance of instrumental reason in medicine. To what extent are we “solving” existential questions of suffering, despair, and loneliness through technical means (such as medication and pathologization) and thereby making existential phenomena diagnoses? [14] What are we unlearning regarding processes rooted in existential, interpersonal, and spiritual competence?

Referring to current debates around practices of assisted dying, which tend to loom between dogmatism and skeptical neutralization, the questions raised above illustrate that assisted dying implies a multitude of fundamental and profound philosophical questions. The critical point is that these are not abstract but implicitly experienced and answered by the practices we perform individually and organize socially. Between the poles of dogmatism and skeptical neutralization, however, philosophical and ethically relevant questions seem to fall into a void and often go unnoticed even within medical ethics support systems for practice.

Thus, based on our exemplary review of basic conceptions surrounding assisted suicide, it is worth to strengthen the consideration and function of ethics consultation in relation to these public realms. As sociologists [15] and ethicists [16] have pointed out decades ago: “at the bedside” (i.e., in general, in immediate decision situations or in the development of organizational guidelines) decisions are made not only with relevance to the persons concerned; in the long term and far more unnoticed, decisions are made about what conception of humanity, of the good life and death, of faith and the afterlife, coexistence, and the relationships between people and nature we are realizing or gradually changing as a society.

Somewhat unexpectedly, but at a second glance not so surprising in fact, there is a widely unrecognized semi‐institutional offer and “place” that seems to attract people with a need and desire to explore and develop critical and ethical considerations related to assisted suicide. This place is a philosophical practice, as elaborated in the following section.

## 4 Philosophical Practice as a Complement to Ethics Consultation

### 4.1 Philosophical Practice as a Movement and Social Institution

“Philosophical practice” can be described as the manifestation of philosophy that aims to revive the maieutics of everyday life and to organize it in various ways. These reach from philosophizing in one‐on‐one settings as a professional offer of philosophical practitioners to philosophizing in the public and with groups as a volunteer social engagement. While these forms carry forward the legacy of ancient philosophy as “a way of life” [8] and “critique of conceptions”, philosophical practice today constitutes a relatively new, semi‐institutionalized offer. It is made by philosophical practitioners and constituted in the facilitation of philosophical thought and discussion, directed at people who want to place their life questions in a broader context and in dialogic engagement with a concrete other [17].

Offers of philosophical practice have carried this label since about 1981, when Gerd Achenbach, a German philosopher, opened a “philosophical practice” as a place of philosophical counseling. Already before, as early as the 1960s, however, similar offers and efforts were made by philosophers to bring philosophy from academia back to the marketplace [18]. Gradually, these efforts turned into a social movement, and from the late 1980s to the early 2000s, “philosophical practice” grew. The inaugural International Conference on Philosophical Practice took place in 1994 in Vancouver. Since then, various international and national associations of philosophical practice have emerged, and a growing, though still modest, body of literature has emerged. More recently, philosophical practice is seeing a growth in publications on approaches and methods [19, 20, 21].

Considering the nature of philosophizing in philosophical practice, current approaches can be characterized by three ideal‐typical styles [22]. These are generally not dogmatically maintained in actual practice, yet each of them exhibits stylistic tendencies in the philosophical dialogue:

- In the hermeneutic style, people visiting a philosophical practice initially share their experiences in a narrative form. The essence of this narrative becomes the focus of the subsequent philosophical conversation. Representatives of this style are, for example, Gerd Achenbach [23] and Anders Lindseth [24].
- In the more analytical style, philosophizing is focused on linguistic precision, logical structure of statements, and rigorous examination of assumptions for their relation to reality. This style often begins with a concrete (philosophical) question posed by the visitor, which is then picked up for further examination. Representatives of this style are, for example, Oscar Brenifier [25], Isabelle Millon, and Elliot Cohen [26].
- The third style takes a more contemplative approach, which tends to take place in group offers. It typically takes the form of the communal contemplation of philosophical texts and uses specific techniques that encourage participants to philosophize from their own philosophical depths, as described by its representative Ran Lahav [27].

Regardless of the styles that philosophical dialogue takes in concrete practice, it is significant for the purposes of this paper that philosophical practice is a publicly available low‐threshold offer of philosophizing about the kind of philosophical questions that trouble us in everyday life. It seems to carry potential as a social institution in which people find support with ethical considerations and a critique of basic conceptions of modernity—involving those surrounding the philosophical question of assisted suicide. This is strongly suggested by insights gained by our research project on philosophical practice in palliative care and hospice work.

### 4.2 Insights From a Research Project Philosophical Practice in Palliative Care and Hospice Work

The ongoing research project philosophical practice in palliative care and hospice work, which involves all the authors of this article, provides preliminary results that illustrate how philosophical practice gives a place to lived concerns around death, dying, and mourning—including (assisted) suicide. In this paper, we focus on insights gained from an interview study with philosophical practitioners (*n* = 29). Amongst other questions, the participants were asked how the topics of “dying, death, and grief” appear in philosophical practice. Participants were interviewed individually or in groups. The full data set consists of 1328 min of audio recording, transcribed for the purposes of qualitative content analysis [28, 29]. Forming broad coding categories that correspond to the interview questions first, the data were then coded inductively by two members of the research team. Two other researchers coded the data in less detail. In two data analysis sessions, the coding was compared to check for reliability and coverage of the research interest. In a final session with the whole research team, the preliminary final code categories and themes were presented and discussed for further enquiry. For the purposes here, we focus on the material that covers participant reports on considerations related to (assisted) suicide. The interview partners are not a vulnerable group and have all given their written consent. The statements do not allow any conclusions to be drawn about specific individuals.

The most relevant finding for this paper is that people seeking philosophical conversation with a philosophical practitioner often do so with an indirect connection to ethical decision‐making situations. The material demonstrates that people often want to develop a position on specific questions in advance (such as assisted dying, the concept of a good death, and related wishes) and engage with a variety of associated questions. Additionally, it is indicated that visitors of philosophical practice show a need to mentally process decisions that have already been made (e.g., the assisted suicide of a family member or consenting to an organ donation). The material also highlights their need to address topics in difficult, ongoing situations that do not directly pertain to the decision at hand but influence it and play a role in the broader horizon of their lives. It thus seems to be the case that, as an emergent social institution, philosophical practice does not intervene with the decision‐making situation concerning typical ethical issues in palliative care to support immediate decision‐making—this role is evidently fulfilled by established ethics structures. Instead, philosophical practice offers a space for addressing implicit, “related” philosophical questions that otherwise find no outlet. It reveals questions that, in a professional context, have no place at all but raise serious and lived philosophical, political, and existential issues. Based on these insights, philosophical practice appears to be a valuable complement to institutionalized ethics consultation.

The following data excerpts illustrate the findings just outlined in the form of the experiences shared by the participating philosophical practitioners of the study. The material covers the kind of questions and stories brought by visitors to the respective philosophical practice in connection with ethical issues.

Example 1 (Interview 14, Pos. 8):

*“There was a difficulty between the rational aspect—that it is good that the parents made this decision themselves—and the emotional aspect—that they left me. And this was not a psychological problem; it was a philosophical problem. It's always very close; it had nothing to do with illness but with the existential and the question, am I allowed to have this feeling.”*

In this passage, the philosophical practitioner reports on a conversation with a guest whose parents ended their lives through assisted dying. The question is not whether the assisted suicide was permissible or good, but rather: What does it mean to be left in this way? What emotions am I allowed to feel?

Example 2 (Group interview 2, Pos. 10):

*“As I listened to you, a few other topics came to mind where people come to me… I just recently had a conversation… in the area of euthanasia… where are the boundaries and one's own stance on it. These are always very, very heated discussions because opinions can vary so much.”*

In this second example, a philosophical practitioner describes a conversation that touched on questions surrounding euthanasia. While some focus of this conversation was on clarifying distinctions, such as assisted suicide versus euthanasia, upon request, the primary focus was on exploring one's own stance and engaging with different perspectives.

Example 3 (Interview 13, Pos. 10):

*“A guest, who had been coming to me for a long time, was dealing with the concrete situation of her brother… who had fallen into a coma and had been on artificial respiration for several months… because no advance directive or similar document was in place. This was… a highly intelligent, reflective person… who had herself grappled with philosophical questions of responsibility at the end of life… questions that we frequently face in connection with the overarching theme of your research project… the experience of personal helplessness.”*

In this final example, the conversation is described to not only address a change in treatment goals but also implicit and fundamental questions of personal responsibility and the meaning of helplessness, along with how to cope with it.

These three examples provide a brief illustration within the scope of this article of the broad spectrum of questions that may be raised within an extended philosophical framework. In all the examples, the primary focus is not on a specific decision or questions of permission but on implicit philosophical questions concerning one's life philosophy, personal understanding of humanity, and concept of good life. For those pre‐decision stages (developing a position, attitude, and related questions about humanity and the good life), the post‐decision stages (what does this mean for life moving forward?), as well as implicit, previously unheard questions, philosophical practice presents itself as a relevant venue. Philosophical practice furthermore appears an appropriate one since the issues that visitors of philosophical practice seek to discuss are not of a psychotherapeutic concern. They have genuine questions that bother them at the deepest levels of the fabric of social and individual ways of life and attitude—and these show to find a social dialogic response in philosophical practice.

## 5 Practical Implications and Conclusions

Looking ahead, we see the following possibilities of departure for institutions of medical ethics and ethics consultation to move toward a connection with philosophical practice. Medical ethics consultations and institutes of medical ethics could collaborate with philosophical practitioners. We deliberately speak of “cooperation” rather than (immediate) “integration” into the team. Why? Because philosophical practitioners, in their diverse and independent forms of practice, represent the public, the community, and the citizen perspective. This enables perspectives to flourish that might otherwise be “trained out” by acclimating to the organization's thought habits. Which forms of cooperation prove to be useful in this context is a matter of social innovation and further research.

Relatedly, the “internal orientation” of medical ethics and ethics consultation (i.e., focusing on training students, employees, developing guidelines within hospitals or specific organizations, and providing case consultations on the ward) could be enriched by an “external orientation” with corresponding activities. Philosophical questions could become subjects of dialogue in numerous public spaces (philosophical cafés, philosophical walks, philosophical counseling, adult education centers, film nights with discussions, etc.). This dialogue would not aim to provide help, solutions, and answers but to encourage free exploration and reflection, alongside social connectedness. This way, institutions of medical ethics (ethics consultation in hospitals and nursing homes) would serve as initiators in the public and in “caring communities”, potentially stimulating a broader social learning process [30]. Philosophical practice thus also makes it possible to balance the tension between “choice and compassion” [31, 32] that characterizes the end‐of‐life care discourse. In addition to the dominant public discourses of good death, which focus on autonomy, individual choice, and “planning” of the end of life, broader images of relational autonomy and compassion can also emerge. Bridges are built between the individual and the collective, the existentially personal and the publicly political.

Turning to philosophical practice itself, we see it as essential that philosophical practitioners working within the medical and care fields not only have philosophical education and training in philosophical practice. Beyond, they need to (a) acquire specialized knowledge in medical ethics, nursing ethics, social work ethics, and related areas of applied ethics; and to (b) thoroughly reflect on the opportunities and limitations of their own philosophical practice—for example, by recognizing if individuals in existential situations who visit their practices might also need professional psychotherapeutic help. Adhering to common ethical standards regarding informed consent, data protection measures for vulnerable situations, and the like is expected and part of a professional ethos within philosophical practice [17, p. 264].

In conclusion, the history of ethics consultation is an impressive success story of providing a space for ethical reflection. However, it is also clear that many philosophical questions remain implicit and placeless within a focus on ethical dilemma situations. Our research suggests that connecting ethical structures with philosophical practice could represent a significant future step for medical ethics. The example of assisted practices of dying, which is not only an issue but also a symptom of contemporary society, has made this especially clear. The link between questions “at the bedside” and those that concern society as a whole became evident: societies' dominant conception of humanity, understanding and practice of social cohesion, and its level of democratization must be brought into focus because the existential questions of individuals represent existential and directional questions for society itself. Philosophical practice could provide the needed democratic space for an open exploration of these deep questions. Before making the connecting step, however, further research, transparency, and experience with philosophical practice in the context of medical ethics are needed.

## Data Availability

The data that support the findings of this study are available from the corresponding author upon reasonable request.

## Endnotes

^1^
The focus of this contribution is on establishing relationships between medical ethics structures and philosophical practice. Remaining within the scope of this paper, we thus cannot elaborate the history and forms of ethics consultation in the health and social systems, nor the underlying understanding of ethics. Instead, we refer to discussions and previous outlines of these questions elsewhere [1, 2]. Note that we also do not speak of “clinical” ethics consultation, as ethics consultation in nursing homes, outpatient care, and the community is also included.
